# VERD: Emergence of Product-Based Video E-Commerce Retrieval Dataset from User’s Perspective

**DOI:** 10.3390/s23010513

**Published:** 2023-01-03

**Authors:** Gwangjin Lee, Won Jo, Yukyung Choi

**Affiliations:** 1 Department of Intelligent Mechatronics Engineering, Sejong University, Seoul 05006, Republic of Korea; 2 Department of Artificial Intelligence, Sejong University, Seoul 05006, Republic of Korea

**Keywords:** computer vision, information retrieval, content-based video retrieval

## Abstract

Customer demands for product search are growing as a result of the recent growth of the e-commerce market. According to this trend, studies on object-centric retrieval using product images have emerged, but it is difficult to respond to complex user-environment scenarios and a search requires a vast amount of data. In this paper, we propose the Video E-commerce Retrieval Dataset (VERD), which utilizes user-perspective videos. In addition, a benchmark and additional experiments are presented to demonstrate the need for independent research on product-centered video-based retrieval. VERD is publicly accessible for academic research and can be downloaded by contacting the author by email.

## 1. Introduction

Image retrieval aims to search the database for images that are similar to a given query image. This technology has been used for automatic checkouts, which scan for products at supermarket cash registers. However, as a result of the recent expansion of the e-commerce market induced by the development of communication technology, research has been conducted to find similar items in online shopping malls.

The goal of the offline datasets [1,2,3,4,5] used for image retrieval in conventional stores is to identify the products at the checkout counter in order to complete automatic payment. These datasets comprise images of products placed on shelves in supermarkets in order to find which items are placed on the checkout desk. Due to the characteristics of this product arrangement, the images in datasets have a uniform background due to the fact that they were filmed in a confined space, although there could be subtle variations in illumination and background depending on the display condition. In addition, they consist of photos taken from the product’s front that clearly depict the brand and its characteristics in order to facilitate automatic checkout.

As previously stated, the growth of the e-commerce market has resulted in the emergence of online datasets [6,7,8,9] used to find similar products in images. These datasets have a more complex background than prior datasets used for automatic checkout. This is because the datasets consist of both images captured by users and uploaded by the sellers to promote the item. The data obtained from the user are realistic, but the product image processed by the seller may include marketing text or other effects. There is a difference in the angle of view, illumination, color, and background between the objects photographed by the actual user and the objects photographed by the seller. These differences make it difficult to identify real objects.

Multimodal online datasets [10,11,12] have appeared to tackle these problems and enable a more sophisticated product search. In contrast to previous studies that relied solely on images, most of the multimodal online datasets contain text and image information that can be used for retrieval, and audio and video information are also being utilized in research [13]. By using extra information from multimodal online datasets, retrieval can be achieved even when images have insufficient information to distinguish products. However, because the datasets were derived from data processed by the seller, they lack the very same data as the user’s search environment. In addition, it is inconvenient that users must provide additional data in addition to image data in a real-world retrieval environment.

**Figure 1:**
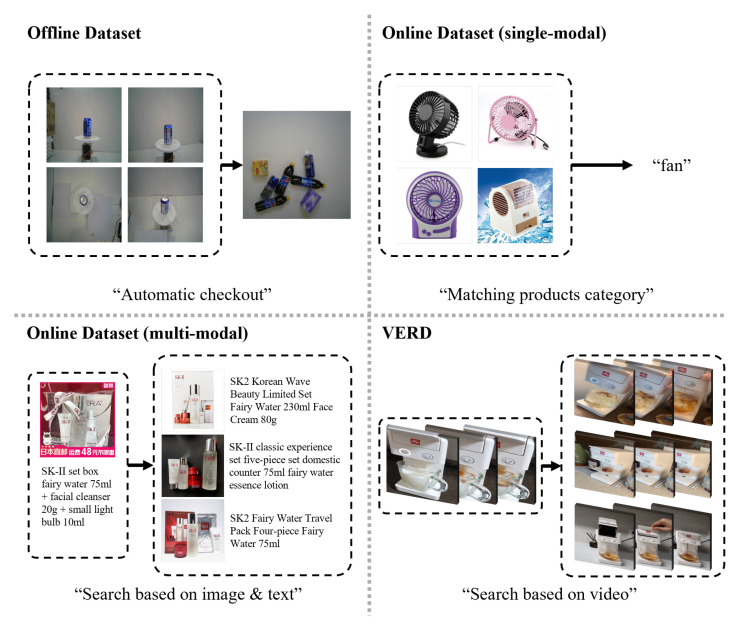
Comparison of datasets related to object-centric retrieval.

To handle the limitations that existing datasets are not comparable to actual search environments and that multimodal datasets do not reduce search complexity, we propose a dataset named Video E-commerce Retrieval Dataset (VERD). Figure 1 shows the difference between the VERD and existing datasets. Traditional datasets collect data based on images, while VERD collects data based on video reviews, which are increasingly popular on the e-commerce platform. This was performed to leverage the idea that video reviews are filmed from a variety of viewpoints and contain a wealth of product-related information. These video reviews were not filmed by sellers, but, rather, by users with the devices that were used to conduct actual searches. Therefore, unlike the data of sellers, filmed in uniform environments, video reviews include a wide variety of backgrounds and camera angles. Based on these attributes, VERD is comparable to the data used in a real-world search, and it aims to conduct retrieval using only the visual information provided by the video, without providing any additional information. Lastly, we present benchmark performance through the existing video retrieval methods [14,15,16] on VERD. We believe that VERD and benchmarks will encourage research on video-based product retrieval.

## 2. Related Work

### 2.1. Datasets

#### 2.1.1. Offline Dataset

Offline datasets are configured to perform tasks such as automatic payment or shop management in conventional grocery stores. Merler et al. [1] proposed the Grozi-120 dataset, which contains images of all products taken in real marketplaces and ideal studios. Jund et al. [2] suggested the Freiburg Grocery dataset, which gathered images from the real-world environments of various shops and apartments to identify various common items, including groceries. Klasson et al. [3] proposed the Grocery Store dataset with hierarchical label information that can combine visual and semantic information on supermarket groceries. Georgiadis et al. [4] suggested the Products-6k dataset, which was created by capturing photos containing product brand names or product descriptions for large-scale product recognition in a supermarket environment. Wei et al. [5] proposed the Retail Product Checkout (RPC) dataset for automatic checkout, which consists of images of objects taken from multiple angles.

#### 2.1.2. Online Dataset

Online datasets, as opposed to offline datasets, comprise data acquired in a varied environment because they collect data uploaded to the e-commerce market. These online datasets can be broadly categorized into two types. The first type of online datasets is a single modality dataset comprising images. Song et al. [6] suggested the Stanford Online Product (SOP) dataset, which has five photos per class but a vast number of classes collected from an e-commerce website. Liu et al. [7] offered the Deepfashion dataset, which contains a variety of images of fashion items, ranging from posed store images to unsupervised consumer photographs. Ge et al. [8] proposed the Deepfashion2 dataset, which includes numerous landmarks and skeletons extracted from fashion-related images. Bai et al. [9] proposed the Product-10k dataset, which consists of photographs of frequently purchased e-commerce product classes across multiple categories, such as food, fashion, and household products.

The second type of online dataset is a multimodal dataset, mainly consisting of text and image data. Corbiere et al. [10] proposed the Dress Retrieval dataset, a noisy image–text multimodal dataset for e-commerce website catalog product descriptions. Chen et al. [11] offered the MEP-3M dataset, which applied hierarchical labels to image–text pair data acquired from Chinese online shopping websites. Zhan et al. [12] proposed the Product 1M dataset containing extensive cosmetic data by gathering textual descriptions of cosmetics and product displays. Dong et al. [13] suggested the M5 product dataset with several modalities, including audio, video, and text, utilizing data uploaded by online retailers.

### 2.2. Methods

#### 2.2.1. Image-Based Retrieval

Traditionally, image-based product retrieval studies [17,18] were conducted in the offline market for applications such as automatic checkout or store management. George et al. [17] proposed a genetic algorithm optimized by multilabel image classification to identify products on shelves. Li et al. [18] proposed the Data Priming Network (DPNet) for automatic checkout to pick reliable samples utilizing the detection and counting collaborative learning strategy during the training process.

In addition, research is extending to include online shopping malls due to the expansion of the e-commerce market. These methods [19,20,21] are typically employed to recommend similar products to users, as well as to locate and recommend similar products, by combining various models that can extract varied product attributes. Shankar et al. [19] introduced VisNet, an end-to-end DCNN architecture comprising deep and shallow networks. Yang et al. [20] and Hu et al. [21] developed a visual search system that uses a reranking mechanism that can be can be applied to large search engines.

#### 2.2.2. Video-Based Retrieval

The majority of research on video-based retrieval focuses on video copy detection for video copy protection and verification, and also content-based video retrieval for video recommendation. These studies can be classified into two categories based on the similarity-calculating method.

The first methods [15,22,23] extract frame-level features, conduct interframe similarity calculations, and then aggregate the results into video-level similarities. Tan et al. [22] proposed a temporal network (TN) using graphs generated by keyframe matching. Chou et al. [23] proposed dynamic programming (DP), which extracts the diagonal pattern from a frame-level similarity map to detect a spatiotemporal pattern. Kordopatis et al. [15] proposed video similarity learning (ViSiL), which employs metric learning combining chamfer similarity to calculate pairwise similarities on an interframe similarity map.

The second methods [14,16] encode video-level features by aggregating frame-level features derived from images and calculating video-level similarity by comparing the obtained features. Kordopatis et al. [14] proposed deep metric learning (DML) utilizing $L_{N}-\mathrm{iMAC}$ [24]. Shao et al. [16] proposed temporal context aggregation (TCA), which utilizes the self-attention mechanism to integrate long-range temporal information between frame-level features.

#### 2.2.3. Multimodal-Based Retrieval

Recently, with the emergence of datasets that support various modalities, studies using various modality information have emerged. Shin et al. [25] proposed e-CLIP, which can be deployed on multiple e-commerce downstream tasks, based on an approach [26] that utilizes both visual and language information. Dong et al. [13] proposed the Self-harmonized Contrastive Learning (SCALE) framework, which unifies the several modalities into a unified model through an adaptive mechanism for fusing features.

## 3. Proposed Dataset

### 3.1. Video Collection

This section discusses the data collection procedure in the Video E-commerce Retrieval Dataset (VERD). We aimed to create a dataset with scenarios resembling those in which consumers look for objects in video. To accomplish this objective, VERD was collected using recently introduced video-based product reviews from online shopping malls (https://shopping.naver.com (accessed on 31 May 2022)).

**Figure 2:**
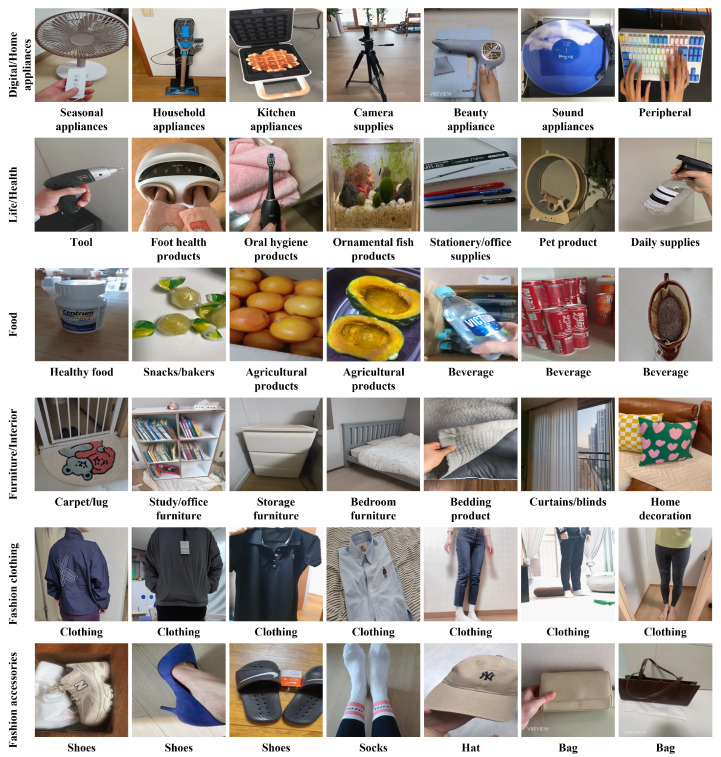
Videos of the hierarchical category of VERD. The category to the left of images represents the Level-0 category, and the category below images represents the Level-1 category.

These product reviews were freely filmed to describe the things that consumers purchased. Due to the various viewpoints, it has a complex background as well as differences in illumination and color. In addition, despite being a review of the same product, the captured area varies according to what the buyer wants to show. As shown in Figure 2, these characteristics allowed us to collect realistic data from the same environment as the user’s search devices.

### 3.2. Annotation Process

This section explains the processing of the dataset. Due to the flexibility of user-uploaded video reviews, we find that reviews are sometimes irrelevant to the product or inadequately depict the product during the data collection section. To address these issues, we conducted a four-step preprocessing procedure to obtain a clean dataset.

The first step is to remove duplicate videos. Occasionally, the same video was reused for many reviews on the e-commerce platform. To eliminate these duplicate videos, Video Duplicate Finder (https://github.com/0x90d/videoduplicatefinder (accessed on 7 July 2022)) was employed. Additionally, visually similar but nonidentical videos were deemed irrelevant and removed.

In a second step, the face-containing video was excluded. We found that in some video product reviews, the user’s face was captured with the product. These reviews contain products, but they are not filmed around the items themselves, making it difficult to identify objects. To filter these videos for object-centric video retrieval, RetinaFace [27] was used to recognize video frames containing faces. If a video had an identifiable face in even a single frame, it was excluded from the dataset.

In the third step, videos captured away from the object’s center were discarded. Typically, this is the case for a long-form review. Long-form reviews provide a comprehensive explanation of the product from the perspective of a product review. However, these reviews contain numerous frames that are irrelevant to the item from the perspective of product search. Therefore, these videos were omitted from the dataset because they did not align with the goal of the dataset collection.

In the final phase, labels were adjusted based on their visual similarity with hierarchical category labels. In fact, videos in the category “coffee” can be divided into a subcategory “capsule coffee” and “cold brew coffee”. These two items were labeled as the same product up to the level of subdivision, although their physical properties were different. Therefore, some labels were reclassified as distinct goods to allow a more detailed search.

Through this annotation process, it was possible to construct a precise dataset with less noise by excluding videos that did not correspond to the data collection goal. In conclusion, VERD includes a total of 41,570 videos and 187 categories.

### 3.3. Hierarchical Category Labeling

Following the annotation process, this section describes the category configuration of the VERD. In the majority of datasets, a label associated with a product relates to a fixed value. This fixed label is inappropriate from the perspective of the retrieval task, which needs to search for related objects. Therefore, we adopted hierarchical category labeling to understand the relationship between products, taking into account the nature of the e-commerce market that sells a wide range of goods.

The hierarchical category labeling that we established is a new definition of product taxonomy. Generally, e-commerce markets employ product taxonomy to facilitate the sale of goods. However, the existing product taxonomy has separate categories for products with similar visual qualities or is unable to distinguish between products within the same category. To overcome these difficulties, we created a new product taxonomy based on whether a product can be visually classified.

**Figure 3:**
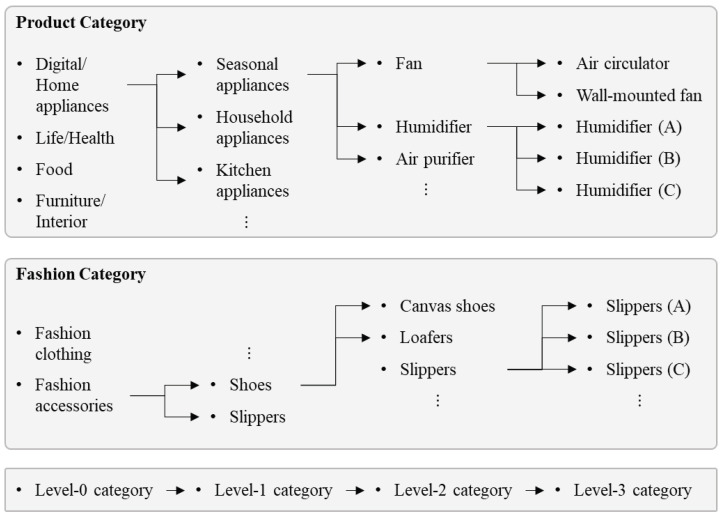
An example of the hierarchical categories of VERD.

The hierarchical category is separated into four levels, whereby the higher the level, the more specific the product classification. From Level-0 to Level-3, there are 6 categories, 44 categories, 119 categories, and 91 categories, respectively. Every video has a hierarchical category with a minimum Level-1 and a maximum Level-3. Figure 3 provides a detailed illustration of hierarchical category. Even though “fan” and “air circulator” have the same Level-2 category, “fan” is subcategorized further for “air circulator”, which works similarly to a fan but differs visually. However, there were occasions in which products in the same class could be visually distinguished from one another. Figure 3 provides another example of this scenario. The “humidifier”, which is designated as a Level-2 category, could be further defined based on how the product performs. In this case, it was modified to add subcategories so that it could be classified into other categories.

### 3.4. Dataset Statistics

This section explains the video statistics of VERD. The dataset contains 41,570 videos. Videos consist of short clips that average 9.8 s. The large majority of videos are under 10 s, and videos under 30 s comprise 94% of the dataset. This demonstrates that most of the videos were filmed around the product rapidly to introduce it.

The dataset can be separated mainly into product-related and fashion-related categories. The product-related category covers the Level-0 categories “digital/home appliances” (10,135), “life/health” (6327), “food” (5754), and “furniture/interior” (1240). Following that, the fashion-related category contains “fashion accessories” (10,283) and “fashion clothing” (7831), for a total of 18,114 videos. This demonstrates that the dataset is dispersed rather equally.

### 3.5. Dataset Characteristics

VERD attempted to construct a dataset that simulates the scenario in which a user conducts an object search through a video. From this perspective, the data can be broadly separated into seller-centric data and user-centric data. In this part, we discuss in detail how user-centric data differ from seller-centric data in terms of the information they may provide.

**Figure 4:**
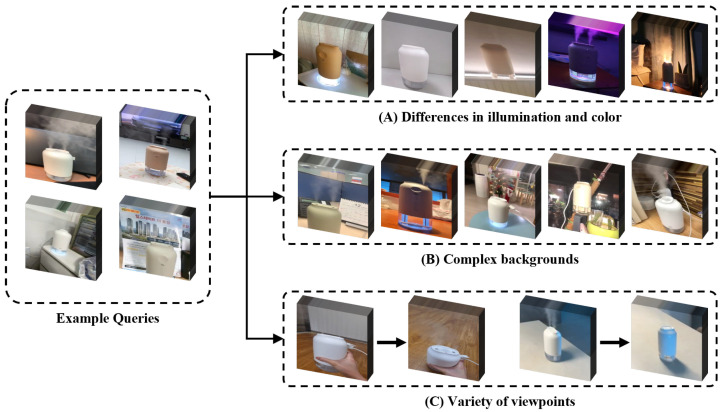
An example of VERD in “Humidifier” category.

**Differences in illumination and color**: Lighting variance can be the most significant difference between the environment presented by the seller and the user. Figure 4A illustrates these attributes. There are instances in which it is difficult to understand the properties of a product due to the surrounding lighting, which is not simply a matter of dark or bright illumination. Even when it was the same product, it occasionally offered various colors. VERD has invested a significant amount of time in collecting these videos so that related products can be identified based on their visual characteristics.

**Complex backgrounds**: Figure 4B shows examples of various backgrounds within the sample videos. In general, seller-centric data exclude a background to emphasize the product. Due to the fact that consumers take shots in a variety of locations, such as their homes and workplaces, multiple items are captured alongside the product. In the videos shown in Figure 4B, it can be verified that the backgrounds are distinctive and do not match. VERD has obtained videos in these varied contexts.

**Variety of viewpoints**: The majority of the information on the page for product sales is taken from the front in order to make the product seem more attractive. However, users do not consider these factors when capturing the product. In order to address this issue, Figure 4C provides examples of videos collected from a variety of perspectives within the dataset. In the example, filming began on the front of the product but was finished by moving the camera upward so that the mechanical part of the product could be seen clearly. In this real-world scenario, including the search for various product parts, the video-based VERD can work effectively.

We illustrate numerous examples of the user-filmed environment by describing Figure 4 and the characteristics of the dataset. They may have a complicated history with irrelevant items and diverse viewpoints. These characteristics suggest that VERD is suitable for real-world scenarios.

## 4. Experiments

### 4.1. Setup

In this section, we propose a benchmark performance with several video retrieval systems. Among these methods, we conducted experiments on DML [14], ViSiL [15], and TCA [16] that published codes. Following the previous approach, the performance was also reported as mean average precision (mAP).

Due to the absence of available training datasets for object-centric video studies, K-fold cross-validation was applied as the evaluation approach. We fixed query videos in the dataset and set K to 5 to split the database. In order to ensure that a sufficient quantity of data is used in the search, the experiment was constructed so that while one fold was used for learning, the remaining fold was used for evaluation.

### 4.2. Benchmark

Table 1 shows benchmark results for existing video retrieval models on VERD. Benchmark experiments were conducted using the authors’ provided code, with only a few hyperparameters modified. All performances were evaluated by choosing the methodology for which the highest performance was reported for each method ($ViSiL_{v}$, $TCA_{f}$, $DML_{late}$).

Benchmark performance was obtained by separating the product category and the fashion category. This is due to the fact that the two categories have different visual qualities. As a result, items in the fashion category have varied shapes based on whether or not they are worn by humans, whereas the visual aspects of products change based on location but the shape of the item does not change. Therefore, the overall performance of the fashion category was deemed to be inferior to that of the product category.

On the other hand, it is noticeable that the performance, in general, is insufficient. This demonstrates that the existing video-to-video retrieval model did not acquire the properties required by object-centric video datasets such as VERD, as it was mainly researched using incident-centric videos. Consequently, the experimental result shows the need for future independent object-centric video retrieval study.

### 4.3. Analysis

#### 4.3.1. Feature Comparison

Most video retrieval methods use frame-level features or video-level features to calculate video similarities. The frame-level feature calculates the similarity between each frame to determine the similarity of the video, while the video-level feature compresses the feature representation of the video to determine the similarity.

Table 2 presents the performance based on the feature difference in TCA [16] to determine the difference between frame-level and video-level feature presentation in object-centric video retrieval. Experiments indicate that the type of feature has a negligible impact on the feature’s performance. This indicates that VERD was taken around an object, allowing the model to understand the expression of the object in the majority of video frames. Moreover, despite the fact that frame-level features perform better in incident-centric video retrieval studies, video-level features are appropriate for e-commerce platforms that need speedy search when performance gaps among feature types are considered.

#### 4.3.2. Modality Comparison

To demonstrate that video clips have a higher volume for visual representation than images, analysis was conducted to compare the performance of image-based and video-based retrieval in Table 3.

The experiment employed the same K-fold cross-validation as Section 4.2; however, in the analysis experiment, only evaluation sets were used identically since there was no training engaged. Moreover, since there was no corresponding dataset for images and videos, a pseudo image dataset was created in VERD for the experiment. This dataset was processed by extracting images from the video’s intermediate frame.

Using the method of [15], a simple video search model was built in order to evaluate the performance of the dataset created in this approach. Similarity was calculated using chamfer similarity with L4-iMAC as a feature.

Table 3 demonstrates that video-based methods consistently outperform image-based methods. This difference in performance is because the video was taken from multiple perspectives, allowing it to be responded to even if the front and side visual characteristics of the product are varied. This means that the video contains more information than the image, as this paper suggests. In the case of existing video search models, where the focus is on incident-centric video retrieval, Table 1 indicates that the performance does not improve significantly, even after training. This demonstrates the necessity for independent research on object-centric video retrieval.

## 5. Conclusions

Object-centric retrieval in the user environment is a major task that can be handled in the expanding e-commerce industry. According to this trend, research on single and multimodal search based on product images emerged, but the challenge was that it was difficult to respond to complex scenarios or that the quantity of data required for a search was massive. Therefore, we propose the Video E-commerce Retrieval Dataset (VERD), comprising videos that have not been utilized in previous studies. We present benchmark performance experiments applying the proposed dataset to existing video search methodologies, and additional experiments indicate the better performance of videos relative to images, demonstrating the need for video-based research.

## Figures and Tables

**Table 1:** Benchmark results of applying VERD to existing video retrieval methods.

Method	Category	Fold					Mean
		1	2	3	4	5	
DML [14]	Product	0.081	0.080	0.087	0.077	0.083	0.082
	Fashion	0.090	0.077	0.097	0.093	0.092	0.090
ViSiL [15]	Product	0.309	0.310	0.311	0.311	0.309	0.310
	Fashion	0.159	0.159	0.158	0.159	0.161	0.159
TCA [16]	Product	0.290	0.292	0.293	0.293	0.294	0.292
	Fashion	0.175	0.182	0.183	0.184	0.184	0.181

**Table 2:** Performance comparison between frame-level feature ($TCA_{f}$) and video-level feature ($TCA_{c}$).

Descriptor	Category	Fold					Mean
		1	2	3	4	5	
Frame-level	Product	0.290	0.292	0.293	0.293	0.294	0.292
	Fashion	0.175	0.182	0.183	0.184	0.184	0.181
Video-level	Product	0.288	0.290	0.290	0.290	0.292	0.290
	Fashion	0.173	0.181	0.182	0.184	0.183	0.181

**Table 3:** Performance evaluation of the VERD using video-based and image-based retrieval.

Method	Category	Fold					Mean
		1	2	3	4	5	
Image-based	Product	0.191	0.192	0.192	0.192	0.192	0.192
	Fashion	0.100	0.100	0.099	0.100	0.100	0.100
Video-based	Product	0.291	0.291	0.292	0.293	0.291	0.292
	Fashion	0.158	0.158	0.157	0.158	0.159	0.158

## Data Availability

The data presented in this study are available on request from the corresponding author. The provided data can be only used for nonprofit purposes.
